# IgG/IgM-coated gut microbiota in schizophrenia: associations with inflammation disease activity

**DOI:** 10.3389/fpsyt.2025.1689069

**Published:** 2026-01-13

**Authors:** Guohao Xu, Ruibin Luo, Ze Wu, Caihong Liu, Haipeng Liao, Junlin Wu, Zhixiang Li, Yinmei Wang, Xi Chen, Yifan Li, Ruihuan Xu

**Affiliations:** 1 The School of Basic Medicine, Beihua University, Jilin, China; 2 Department of Clinical Laboratory, Second Affiliated Hospital, School of Medicine, The Chinese University of Hong Kong, Shenzhen & Longgang District People’s Hospital of Shenzhen, Shenzhen, Guangdong, China; 3 Department of Clinical Laboratory, Longgang Central Hospital of Shenzhen, Shenzhen, Guangdong, China; 4 Department of Clinical Laboratory, Longgang District Chronic Disease Prevention and Control Hospital, Shenzhen, Guangdong, China; 5 School of Medicine, Chinese University of Hong Kong, Shenzhen, China; 6 School of Medicine, The Jiaying University, Meizhou, Guangdong, China; 7 Mental Health Department, Second Affiliated Hospital, School of Medicine, The Chinese University of Hong Kong, Shenzhen & Longgang District People’s Hospital of Shenzhen, Shenzhen, Guangdong, China; 8 Department of Clinical Laboratory, Seventh People’s Hospital of Dongguan City, Dongguan, China

**Keywords:** schizophrenia, immunoglobulin M, immunoglobulin G, fecal microbiota transplantation, inflammation

## Abstract

**Background:**

While immunoblobulin A(IgA) dominates gut mucosal immunity, the roles of immunoglobulin M (IgM) and immunoglobulin G (IgG) in host-microbiota interactions remain poorly characterized, particularly in schizophrenia (SCZ). Although gut dysbiosis and immune activation have been implicated in SCZ,the contribution of IgG/IgM-coated gut microbiota to disease associated inflammation and behavioral alterations remains unknown.

**Methods:**

We recruited six patients with SCZ, six with other psychiatric disorders (OPD) and six age- and sex- matched healthy controls. IgG/IgM-coated gut microbiota were isolated from 100 mg fecal samples via magnetic-activated cell sorting (MACS) and profiled by 16S rRNA sequencing. A pilot an IgG/IgM-coated fecal microbiota transplantation (FMT) using anaerobically cultured human intestinal microbiota was conducted in mice to assess the effects on gut pathology, peripheral immunity, and behavior. The percentage of neutrophil granulocyte in peripheral blood was quantified microscopically, and statistical analyses were performed using one-way ANOVA in GraphPad Prism 8, with (p < 0.05.

**Results:**

The proportions of IgM-coated bacteria was significantly higher in patients with SCZ than in healthy controls (p<0.05), with enrichment of Rhodococcuss, Shigella, Clostridium and Streptococcus. Mice receiving a mixture of high-IgM-coated intestinal bacteria mixture showed reduced depletion of peripheral neutrophils, mild colon shortening, and mucosal inflammation compared with those receiving low IgM-coated or uncoated bacteria. In contrast, high IgG-coated bacteria, enriched in *Rhodococcuss*, *Acinetobater* and *Pseudomonas*, decreased in SCZ, but induced similar inflammatory gut changes. No IgG- nor IgM- induced anxiety-like behavior were detect in the mice.

**Conclusions:**

Our findings reveal that IgG/IgM-coated intestinal microbiota display distinct immunoreactive microbiota signatures associated with SCZ. These coated communities promote gut inflammation without inducing anxiety-like behavior, highlighting their potential as novel biomarkers of SCZ-associated immune dysregulation and as targets for personalized therapeutic strategies.

## 1 Introduction

Schizophrenia (SCZ) is a serious mental disorder characterized by hallucinations, delusions, impairment, and cognitive dysfunction, and is ranked as the third leading cause of disability globally (1). Although the precise etiology remains elusive, both genetic predisposition and environmental factors are known to contribute significantly to its development (2, 3). Genome-wide association studies (GWAS) have demonstrated that the predictive power of known genetic variants for individual disease risk has progressively increased, accounting for approximately 20% of disease heritability. Nevertheless, this level of accuracy is insufficient to meet the clinical requirements (4). Emerging evidence shows that gut microbiota can influence brain function and play a significant role in mental illnesses such as SCZ. A growing body of research has demonstrated the pivotal role of the “microbiota-gut-brain” axis in the pathogenesis of mental disease, which includes bidirectional communication between the central nervous system and gut microbiota through neural, hormonal and immune pathways (5).

Notably, immune dysregulation has emerged as a key feature in the pathophysiology of SCZ. Multiple studies have reported elevated IL-6, IL-8, IL-1βand TNF-αlevels of pro-inflammatory cytokines, and revealed associations between these cytokines and specific gut microbiota (6–10). Elkjaer et al. showed that immune scores, including these pro-inflammatory cytokines, linked to disease progression in severe mental disorders (10). And Kim et al. also found that a gluten-free diet modulates gut-related immunity and inflammation in gluten-sensitive patients, leading to a reduction in oxidative stress and an improvement in intestinal symptoms (7). Existing studies have shown that, Chinese herbal medicines, compound prescriptions and Chinese patent medicines, can play potential therapeutic effects on cerebral ischemia and liver injury by regulating intestinal flora, reducing neuroinflammation and oxidative stress (11–13). However, the mechanisms by which immune responses to the gut microbiota influence SCZ development remain poorly understood.

In gut mucosal immunity, immunoglobulin A (IgA) serves as the primary defense mechanism, maintaining microbial homeostasis and protecting against pathogens (14, 15). Although IgA dominates mucosal immunity, IgM and IgG are also present, but their interactions with microbiota remain largely uncharacterized. Both IgA and IgM are locally produced by gut-associated lymphoid tissue B cells and are secreted via the polymeric Ig receptor-IgA as dimers and IgM as pentamers (16). Interestingly, about 3-4% of lamina propria B cells constitutively express IgG, with plasmablast frequencies increasing markedly during inflammation (16, 17), potentially linking IgG+ B cells to disease states. Yu Wu et al. also found that significantly enriched bioprocesses concerning the immunoregulatory genes with differential expressions of IgG binding according to GO and KEGG analyses in SCZ (18).

Among immunoglobulins and the microbota, a pivotal study found that approximately 36% of the gut microbiota is coated by IgA under physiological conditions, increasing to 69% during inflammation (19). Through this targeted activity, along with other functions such as immune exclusion, IgA plays a key role in maintaining intestinal homeostasis (20, 21). Current research on IgA-coated microbiota has focused predominantly on inflammatory bowel diseases (22–25). In our unpublished studies, we observed that IgA-coated bacteria induce behavioral abnormalities in mice, along with colonic mucosal necrosis and chronic inflammatory cell infiltration. These pathogenic responses are predominantly T cell-dependent, whereas T cell-independent responses are typically associated with symbiotic bacteria.

Given its structural similarities to IgA, IgM is produced in response to luminal microbial epitopes sampled by dendritic cells that share IgA^’^s microbiota-binding patterns. Recent findings indicate that sIgM can also coat a portion of the gut microbiota along with sIgA (26, 27) suggesting a previously unrecognized role in shaping the gut microbiome and maintaining gut homeostasis, even though its precise function remains largely unknown (28). In contrast, IgG is the most common antibody in circulation but can also be transported into the gut via the neonatal Fc receptor, and is not produced continuously in response to common gut antigens (29). Dysregulated IgG responses have also been shown to contribute to mucosal inflammation. Previous studies have shown that the proportion of IgA- and IgG-coated bacteria in the intestines of patients with IBD is elevated (30, 31). However, the roles of IgM- and IgG-coated microbiota (IgM-SEQ and IgG-SEQ, respectively) in immune discrimination remain poorly understood.

To address this knowledge gap, we employed magnetic-activated cell sorting (MACS) and 16S rRNA sequencing to profile IgG/IgM-coated microbiota in SCZ patients, other psychiatric disorders, and healthy controls. Furthermore, we conducted a fecal microbiota transplantation (FMT) study using anaerobically cultivated human intestinal microbiota to evaluate its impact on intestinal pathology and behavior in a murine model. This study investigation provides novel insights into how IgM-and IgG-microbiota interactions may contribute to SCZ pathogenesis, paving the way for development of targeted therapeutic strategies against IgG/IgM-coated microbiota.

## 2 Methods

### 2.1 Ethical approval

The study protocol was approved by the Institutional Review Board of the Second Affiliated Hospital, School Medicine, The Chinese University of Hong Kong, Shenzhen (approval no. 2024001). All animal procedures were conducted in accordance with protocols approved by the Ethical Committee of Chinese University of Hong Kong, Shenzhen and the Second Affiliated Hospital (approval no. AP-2023012).

### 2.2 Patients

Fresh stool samples were collected from patients with SCZ, patients with other psychiatric disorders (OPD), including depressive disorder, bipolar disorder and organic mental disorder, and healthy controls (HC). All SCZ diagnoses were confirmed by two senior psychiatrists using the Structured Clinical Interview for DSM-IV (SCID). Participants were recruited from the Second Affiliated Hospital, School of Medicine, Chinese University of Hong Kong, Shenzhen, China. Prior to sample collection, all participants were instructed to maintain their usual diet and avoid alcoholic beverages for 48 h. Inclusion criteria were as follows: a primary diagnosis of SCZ, OPD, or classification as a healthy control, and age between 18 and 50 years. Exclusion criteria included the presence of any major physical comorbidities (e.g., inflammatory bowel disease, diabetes, autoimmune disorders), co-occurring other mental disorders or a history of illicit drug use, or the intake of antibiotics, probiotics, or prebiotics within the four weeks preceding sample collection.

Fresh stool samples were self-collected using a provided sterile container and return it to the clinic within 2 hours. Each sample was thoroughly homogenized, and aliquots of approximately 100 mg were made into sterile cryotubes and immediately frozen at −80 °C until processing.

### 2.3 Fecal sorting of IgG+/IgM+ and IgG-/IgM-bacteria

IgG/IgM-coated bacteria were isolated using an optimized protocol adapted from Palm et al. (21). Briefly, 100 mg aliquots of frozen human fecal samples were homogenized in FastPrep Lysing Matrix D tubes containing ceramic beads (Miltenyi Biotec, Germany) with 1 mL of ice-cold phosphate-buffered saline (PBS) per 100 mg of fecal material. Following a 1-h incubation on ice, samples were mechanically homogenized for 5 s and subjected to differential centrifugation: first at 50 × g for 15 min at 4°C to remove large particles, then at 8,000 × g for 5 min at 4°C to pellet the bacteria. The bacterial pellets were washed with PBS containing 1% (w/v) bovine serum albumin (BSA; Beijing 4A Biotech, China) and resuspended in staining buffer (PBS + 0.5% BSA), with 20 μL aliquots saved as pre-sort controls for subsequent 16S rRNA sequencing analysis.

For antibody staining, bacterial suspensions were blocked with staining buffer containing 20% normal mouse serum (Jackson ImmunoResearch, USA) for 20 min on ice, and incubated with 2% phycoerythrin(PE)-conjugated anti-human IgG/IgM antibodies (Miltenyi Biotec Germany) in staining buffer for 30 min on ice in the dark. After three washes with staining buffer, the labeled bacteria were incubated with 50 μL of Anti-PE MACS beads (Miltenyi Biotec Germany) in 1 mL staining buffer for 15 min at 4°C.

Magnetic-activated cell sorting (MACS) was performed using MS columns according to the manufacturer’s protocol, with the negative fraction (IgG/IgM- bacteria) collected in the flow-through and the positive fraction (IgG/IgM+ bacteria) eluted after the column was removed from the magnetic field. The IgG/IgM+ fraction was washed with PBS containing 1% BSA, and approximately 2 × 10^6^ sorted bacteria per sample were pelleted by centrifugation at 10,000 × g for 5 min at 4°C. All fractions (pre-sort, IgG/IgM+, and IgG/IgM-) were stored at -80°C for subsequent 16S rRNA sequencing and additional analyses.

### 2.4 Culturing of human fecal bacteria and generation of personalized microbiota culture collections

Cultivation of human fecal bacteria and generation of personalized microbiota culture collections were performed as described by Goodman et al. (32) with modifications. Serial dilutions of fecal samples from six patients with schizophrenia (SCZ) were plated on three selective media: CDC anaerobic 5% sheep blood agar (with and without antibiotic supplementation using kanamycin and vancomycin; Sigma-Aldrich, USA), and gut microbiota medium (GMM) agar (Zhenzhou Autobiotech Co., Ltd, China). From each patient, 100−200 distinct colonies were isolated and individually cultured in GMM under anaerobic conditions for five days to establish comprehensive culture collections.

### 2.5 Assembly of IgG+/IgM+ and IgG-/IgM- consortia and colonization of germ-free mice

For consortium assembly, bacterial strains were selected from these culture collections based on their IgA coating indices (ICI) determined by IgA-SEQ analysis. The IgG+/IgM+ consortium was composed of strains exhibiting high coating levels (ICI > 10) in donor SCZ patients while showing minimal coating in healthy controls. Conversely, the IgG-/IgM- consortium included strains with low coating indices (ICI < 1) in their source patients, which were similarly uncoated in both healthy controls and patients with other psychiatric disorders. Selected strains were cultured separately in GMM for four days before being combined in defined proportions to create the final consortia. Germ-free C57BL/6 mice, housed individually in flexible film isolators, were then colonized via oral gavage with 100 μL of the appropriate bacterial consortium (approximately 1×10^9^ CFU per dose) to establish the experimental microbiota.

### 2.6 DNA extraction, polymerase chain reaction amplification, and Illumina miSeq sequencing

Total genomic DNA was extracted from the bacterial samples using the Multiplex PCR Sample Preparation Kit (Vazyme, Nanjing, China) according to the manufacturer’s protocol. All extraction procedures were performed under sterile conditions to prevent contamination. Following extraction, DNA samples were immediately quantified using a NanoDrop NC2000 spectrophotometer (Thermo Fisher Scientific, USA) to assess concentration (ng/μL) and purity (A260/280 ratio >1.8), followed by quality verification through 1% agarose gel electrophoresis to confirm DNA integrity (fragment size >10 kb) and absence of degradation. All DNA extracts meeting quality control thresholds (minimum concentration 20 ng/μL, A260/280 ratio 1.8−2.0) were aliquoted and stored at -20°C in TE buffer (10 mM Tris-HCl, 1 mM EDTA, pH 8.0) until subsequent molecular analyses.

### 2.7 16*S* rRNA gene sequence analysis

The V3−V4 hypervariable region of bacterial 16S rRNA genes was amplified using barcoded primers 338F (5’-ACTCCTACGGGAGGCAGCA-3’) and 806R (5’-GGACTACHVGGGTWTCTAAT-3’), with unique 7-bp barcodes incorporated for sample multiplexing. PCR reactions (25 μL total volume) contained 5 μL of 5×reaction buffer, 0.25μL of FastPfu DNA polymerase (5 U/μL), 2 μL of dNTP mixture (2.5 mM each), 1 μL each of forward and reverse primers (10 μM), 1 μL of template DNA (~20 ng), and 14.75 μL of nuclease-free water. Amplification was performed under the following thermal cycling conditions: initial denaturation at 98°C for 5 min; 25 cycles of denaturation (98°C, 30 sec), annealing (53°C, 30 sec), and extension (72°C, 45 sec); followed by a final extension at 72°Cfor 5 min. The resulting amplicons were purified using VAHTS™ DNA clean beads (Vazyme, China) and quantified using the PicoGreen fluorescence assay (Invitrogen, USA). Equimolar pools of purified amplicons were prepared and subjected to paired-end sequencing (2×250 bp) on an Illumina NovaSeq platform using NovaSeq 6000 SP reagents (500 cycles), with an average sequencing depth of 50,000 reads per sample, ensuring robust microbial community analysis.

### 2.8 Animal experiments

All animal procedures were conducted in accordance with protocols approved by the Ethical Committee of the Chinese University of Hong Kong, Shenzhen, and the Second Affiliated Hospital (approval no. AP-2023012). Six-week-old male C56BL/6J mice, bred under specific pathogen-free conditions at the Experimental Animal Research Center of the Chinese University of Hong Kong, Shenzhen, were housed in flexible film gnotobiotic isolators throughout the study. Animals were maintained in a controlled environment (temperature: 20 ± 2°C; humidity: 50 ± 10%) with a 12-h light/dark cycle and were provided with autoclaved food and water ad libitum.

To prepare for microbiota transplantation, the mice received a broad-spectrum antibiotic cocktail consisting of ampicillin (1 mg/mL), vancomycin (0.5 mg/mL), neomycin sulfate (1 mg/mL), and metronidazole (1 mg/mL) (33), administered via oral gavage daily for seven days. Following antibiotic pretreatment, animals were randomly assigned to experimental groups (n=8−10 per group) and received daily oral gavage of 200 μL of either (1) IgG+ microbiota, (2) IgG- microbiota, (3) IgM+ microbiota, (4) IgM- microbiota, or (5) saline control for 14 consecutive days.

Behavioral assessments were performed after acclimation. For the open field test (OFT), individual mice were placed in the corner of a 40×40 cm arena and allowed to explore freely for 5 min following a 1-minute acclimation period. Locomotor activity (total distance traveled) and anxiety-like behavior (percentage of time spent in the central 25% area) were automatically quantified using the VisuTrack system (Xinruan Information Technology, China).

Spatial working memory was evaluated using a Y-maze test consisting of three identical arms (45×10×29 cm) arranged at an angke of 120°. Mice were placed at the end of one arm and allowed to explore freely for 5 min. Spontaneous alternation behavior was defined as consecutive entries into all three arms without repetition. The percentage of spontaneous alternation was calculated formulas follow: [Number of sequential arm entries comprising all three arms/(Total arm entries - 2)] × 100%. Between each trial for each mouse, the maze was thoroughly cleaned with 70% ethanol to eliminate any residual odor cues.

All behavioral sessions were video-recorded and analyzed by investigators blinded to treatment allocation using automated tracking software (VisuTrack, version 3.0; Shanghai Xin Luan MDT Infotech LTD, China). Animals were acclimated to the testing room for at least 60 min prior to behavioral assessment to minimize stress-related effects.

### 2.9 Histology

Two weeks after fecal microbiota transplantation (FMT), C56BL/6J mice were humanely euthanized by excessive CO2 inhalation. Briefly, mice were placed in individually ventilated cages (IVCs), the cage lid was secured, the CO2 tube was connected, and CO2 was introduced into the chamber at a rate of 10%−30% of the chamber volume per minute to fill the cage. Once the mice have lost consciousness and motor function, the gas was gradually increased without the maximum flow rate not exceeding 0.5 kPa. After confirming the absence of movement, breathing, and the presence of fixed, dilated pupils, CO2 flow was stopped, and the animals were observed for an additional 2 min to ensure death.

Entire colonic segments were immediately excised, measured and fixed in Bouin’s solution for 24 h at room temperature. Following standard dehydration and clearing procedures, tissues were embedded in paraffin blocks, sectioned at 5 μm thickness stained with hematoxylin and eosin (H&E) using an automated stainer (Leica ST5020). Slides were evaluated by a board-certified veterinary pathologist blinded to the experimental groups. Histopathological scoring (0−4 scale) assessed multiple four parameters: (1) epithelial integrity, (2) inflammatory cell infiltration, (3) crypt architecture, and (4) presence of ulceration or abscesses.

At the time of tissue collection, peripheral blood samples were obtained via cardiac puncture and processed within 3 h of collection. Blood smears were prepared using Wright’s staining method (GE Healthcare, USA) and examined under an Olympus CH20 light microscope (Tokyo, Japan) at 400× magnification. Differential leukocyte counts (neutrophils, lymphocytes and monocytes) were performed by an experienced hematology technician blind to treatment assignments, with a minimum of 100 cells counted per slide to ensure statistical reliability. All histological and hematological analyses followed standardized protocols to maintain consistency across samples.

### 2.10 Statistical analysis

All statistical analyses and data visualizations were performed using GraphPad Prism 8 (GraphPad Software, CA,USA), with supplementary calculations conducted in Microsoft Excel 2019 (Microsoft Corporation, WA,USA). Continuous variables were first assessed for normality distribution using the Kolmogorov−Smirnov test with the Dallal−Wilkinson−Lillie correction for p-values. Normally distributed data were expressed as mean ± standard deviation (SD), whereas non-normally distributed variables were presented as medians with interquartile ranges (IQR). Between-group comparisons were analyzed using one-way analysis of variance (ANOVA) followed by Tukey’s *post-hoc* test for multiple comparisons when the assumption of equal variances was satisfied (as confirmed by Bartlett’s test). For all analyses, p < 0.05 was considered statistically significant.

## 3 Results

### 3.1 IgM-SEQ identifies highly IgM-coated members of the intestinal microbiota

**Figure 1:**
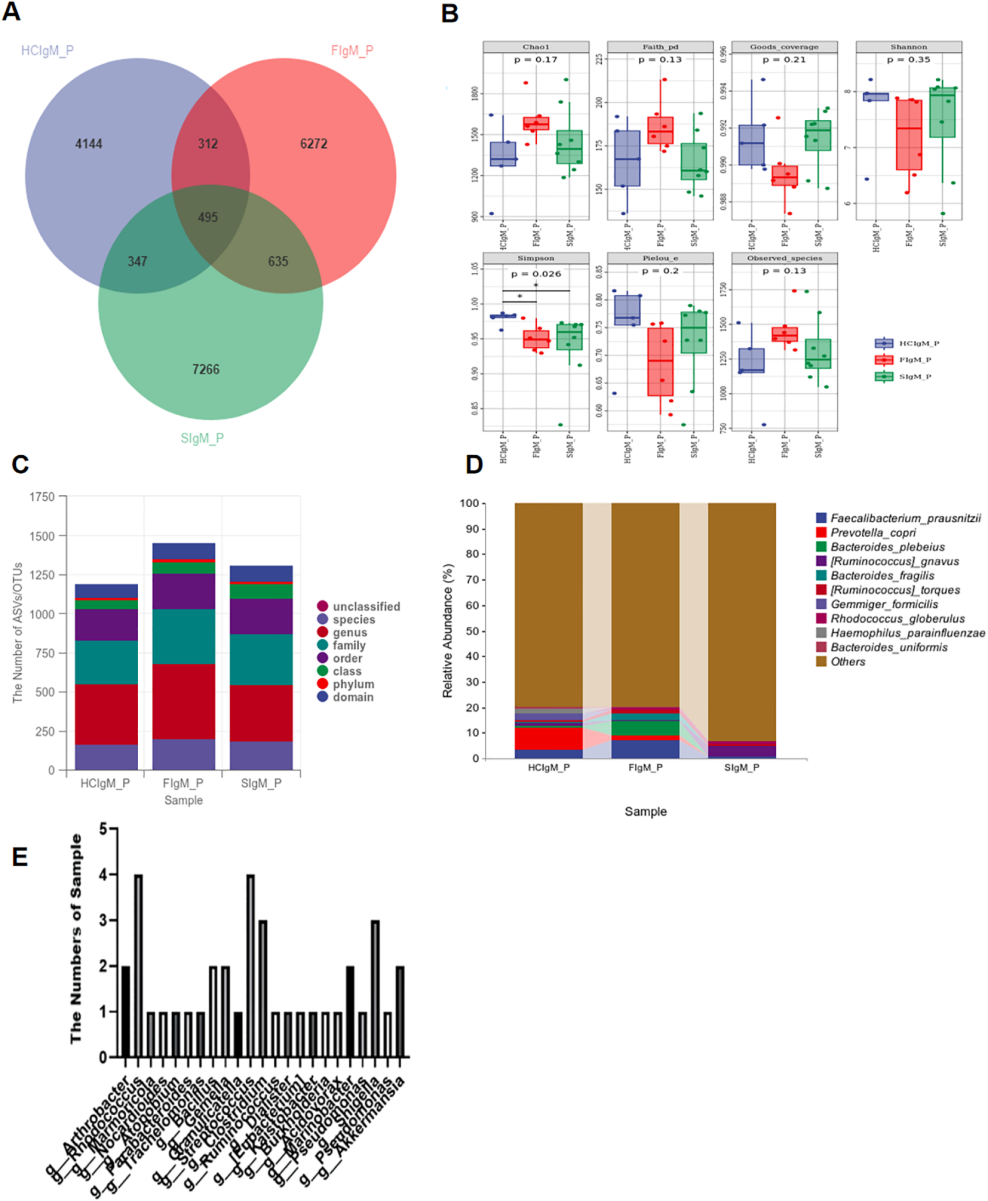
IgM-SEQ identifies highly IgM-coated members of the intestinal microbiota. **(A)** Venn diagram showing the distribution of highly IgM-coated bacterial species among healthy, patients with other psychiatric disorders (OPD), and patients with schizophrenia (SCZ). The schizophrenia group (SIgG P) exhibited the highest of highly IgM-coated bacterial species compared with the other two groups. **(B)** Altered composition of gut microbiota composition in patients with schizophrenia. All data was obtained from the discovery phase, gut highly IgM-coated microbiota richness (Chao 1 and observed species), evolutionary (Faith’s PD) and evenness (Pielou’s evenness) did not differ between SCZ and the other two groups, (p > 0.05, Wilcoxon rank-sum test), while the Simpson diversity index was significantly lower in SCZ than in healthy individuals (p < 0.05). **(C, D)** Relative abundance of bacterial taxa at the phylum and genus levels in the SCZ, OMD, and HC groups. **(E)** Distribution of gut bacteria with an IgM-coated index (ICI) >10 in patients with schizophrenia. *P <0.05.

The results revealed distinct distribution patterns of IgM-coated bacterial species among healthy controls (HC), outpatient department subjects (OPD), and patients with schizophrenia (SCZ), as illustrated in the Venn diagram (**Figure 1A**). Specifically, the SCZ group exhibited the greatest number of bacterial taxa with elevated IgM coating (SIgM P) compared with the other two groups. However, analysis of the overall gut microbiota composition showed no significant differences between the SCZ and control groups in terms of richness (Chao 1 and observed species), evolutionary diversity (Faith-PD), or evenness (Pielou’s evenness) (p > 0.05, Wilcoxon rank-sum test). Notably, the Simpson diversity index was significantly lower in SCZ patients than in healthy controls (p < 0.05)(**Figure 1B**). At the phylum level, no differences in relative abundance were observed between SCZ and HC (**Figures 1C, D**). In contrast, genus-level analysis identified significant alterations in the SCZ group, including decreased abundances of *Faecalibacterium prausnitzii, Prevotella copri*, *Bacteroides plebeius* and *Bacteroides fragilis* along with an increased abundance of *Ruminococcus gnavus* compared with both the OPD and HC groups. The most highly IgM-coated gut bacterial genera ((ICI) >10) were mainly *Rhodococcus*, *Shigella, Clostridium, and Streptococcus* (**Figure 1E**). Overall, these findings indicate that although overall microbial diversity and structure remain largely unchanged in SCZ, specific genus-level imbalances may contribute to the disease phenotype.

### 3.2 IgG-SEQ identifies highly IgG-coated members of the intestinal microbiota

**Figure 2:**
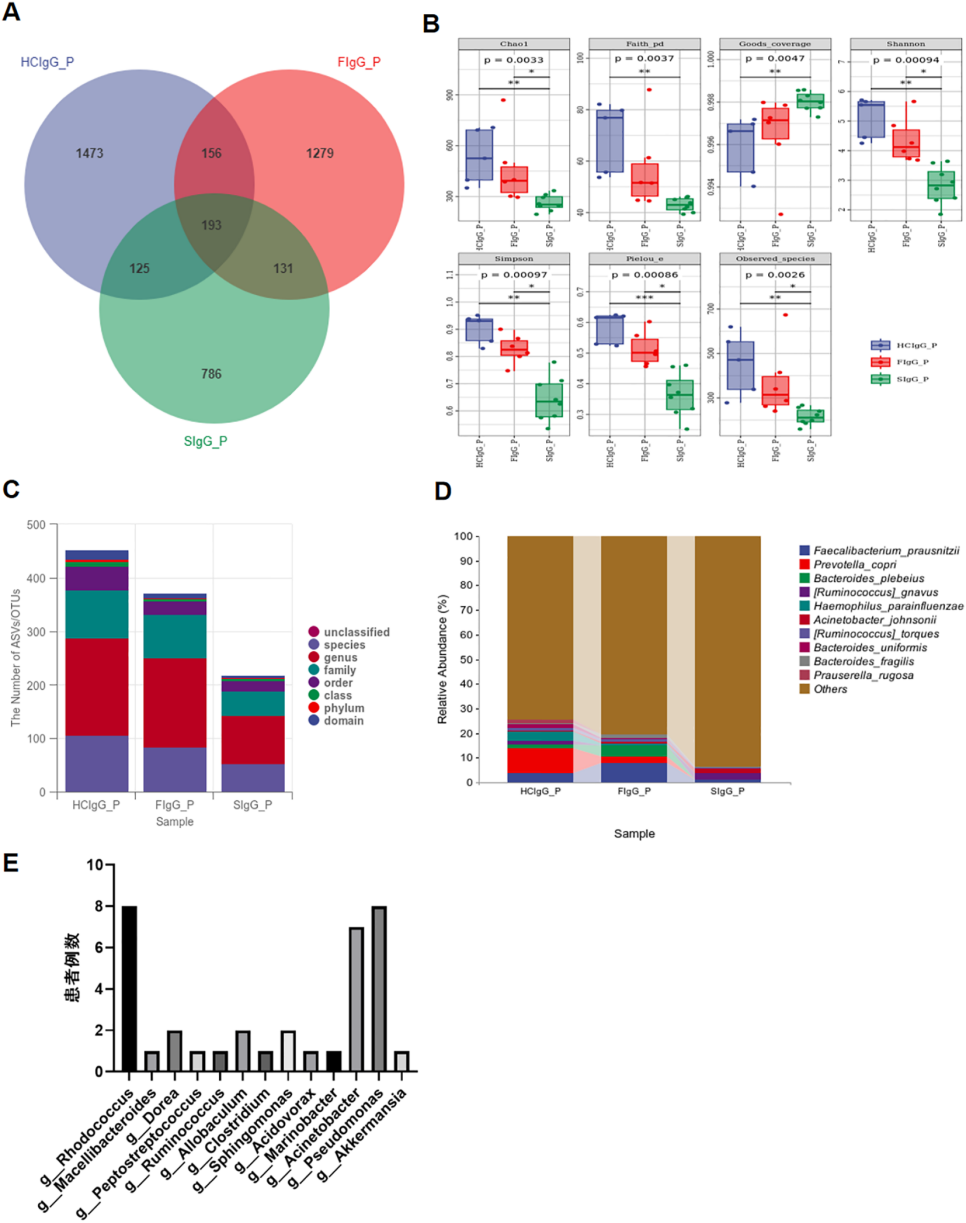
IgG-SEQ identifies highly IgG-coated members of the intestinal microbiota. **(A)** Venn diagram depicting the distribution of highly IgG-coated bacterial species among healthy controls(HC), patients with other psychiatric disorders (OPD), and patients with schizophrenia(SCZ). Bacterial taxa showed the lowest levels of highly IgG-coated bacterial species in the schizophrenia group (SIgG P) than other two groups. **(B)** Altered composition of gut microbiota composition in patients with schizophrenia. All data was obtained comes from the discovery phase: a) gut highly IgG coated microbiota richness (Chao 1 and observed species), diversity(Shannon and Simpson), phylogenetic diversity (Faith’s PD), and evenness(Pielou’s evenness) were all significantly lower in SCZ than in the other two groups (p < 0.01, Wilcoxon rank-sum test); b) gut highly IgG coated microbiota Goods coverage was significantly higher in SCZ than in the other two groups (p < 0.01,Wilcoxon rank-sum test). **(C, D)** Relative abundance of taxa at the phylum and genus levels in the SCZ, OMD, and HC groups. **(E)** Distribution of gut bacteria with an IgG-coated index (ICI) > 10 in patients with schizophrenia. *P < 0.05, **P < 0.01, ***P < 0.001.

The experimental results revealed distinct patterns of the highly IgG-coated gut microbiota among HC, OPD patients, and SCZ patients. As illustrated in the Venn diagram (**Figure 2A**) the SCZ group exhibited the fewest highly IgG-coated bacterial species compared with the other two groups. Further analysis of the gut microbiota composition (**Figure 2B**) revealed significant alterations in patients with SCZ.

In the discovery phase, the richness of the highly IgG-coated microbiota, measured by Chao 1 and observed species indices, was significantly reduced in the SCZ group relative to the HC and OPD groups (p < 0.01, Wilcoxon rank-sum test). Similarly, diversity metrics (Shannon and Simpson indices), phylogenetic diversity (Faith’s PD), and evenness (Pielou’s evenness) were all markedly lower in patients with SCZ (p < 0.01). Conversely, Goods coverage, an indicator of sequencing depth, was significantly higher in the SCZ group than in the other cohorts (p < 0.01). These findings indicate a substantial depletion and altered compositional restructuring of the IgG-targeted gut microbiota in individuals with schizophrenia.

From the phylum to the genus level,relative abundance analysis of IgG-coated gut bacteria revealed significant differences between patients with SCZ compared with HC and OPD. Specifically, the SCZ group exhibited a marked reduction in several bacterial species, including *Faecalibacterium prausnitzii*, *Prevotella copri*, *Bacteroides plebeius*, *Haemophilus parainfluenzae*, and *Acinetobacter johnsonii* (**Figures 2C, D**). In contrast, *Ruminococcus gnavus* abundance was significantly elevated with SCZ. The most highly IgG-coated gut bacterial genera ((ICI) >10) were *Rhodococcus, Acinetobacter and Pseudomonas* (**Figure 2E**). Together, these findings suggest a distinct IgG-targeted microbial signature in schizophrenia, characterized by depletion of potentially beneficial or commensal species and enrichment of potential pathobionts.

### 3.3 SCZ-associated IgG+/IgM+ bacteria induced colon pathology in C56BL/6J mice

**Figure 3:**
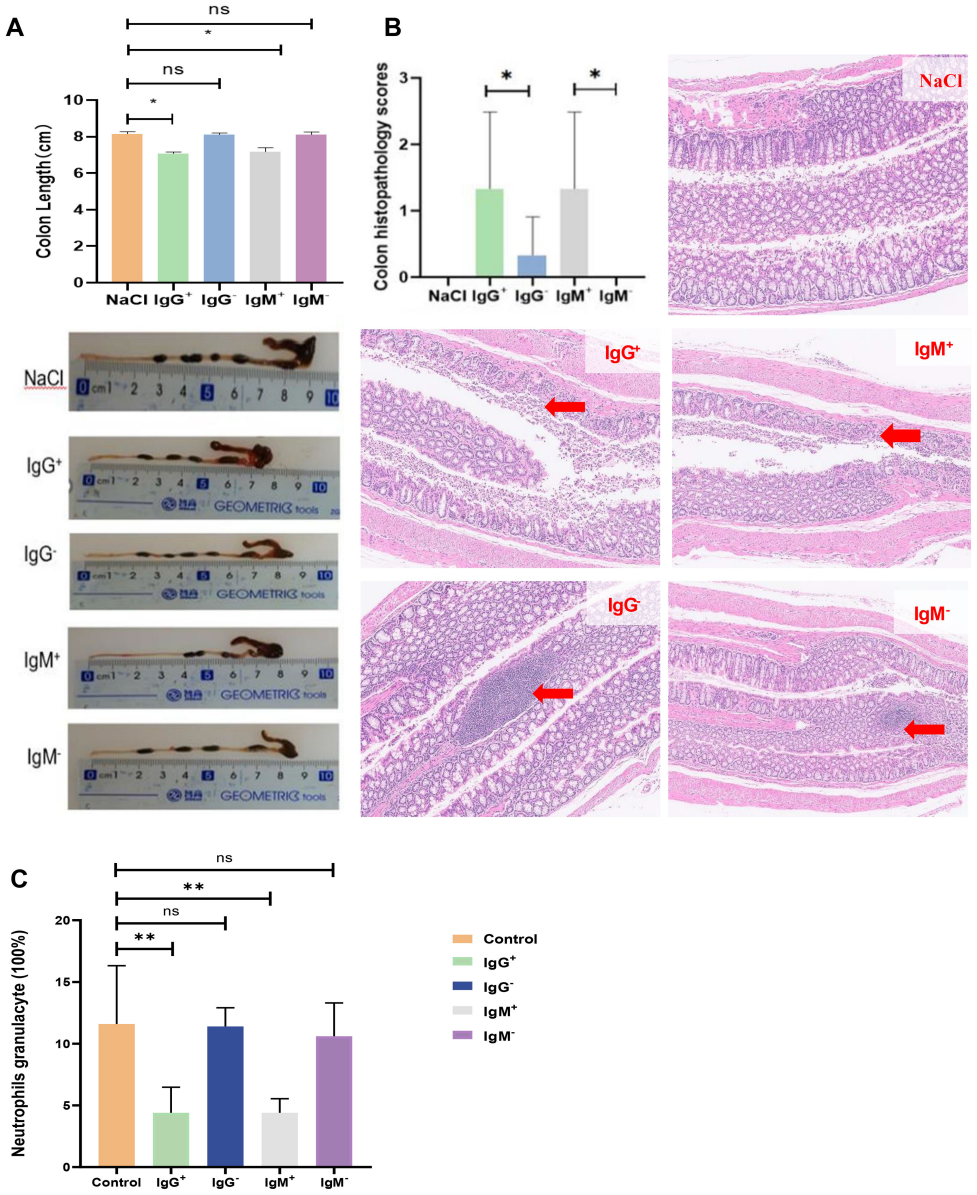
SCZ-associated IgG+/IgM+ bacteria induced colon pathology in C56BL/6J mice. **(A)** Colon length following gavage with SCZ-associated IgG+/IgM+ bacteria. ****p < 0.0001 (unpaired Student’s t-test). **(B)** Representative pathology and hematoxylin and eosin (H&E) stained colons from mice gavaged with SCZ-associated IgG+/IgM+ bacteria. Representative histological images of mice receiving IgG+/IgM+ exhibited extensive inflammation, crypt abscesses, epithelial loss, and ulceration, whereas mice receiving IgG-/IgM-induced mice showed no signs of Inflammation. Histopathological scores were assigned as follows: 0, intact colonic architecture with no acute inflammation or epithelial injury; 1, focal minimal acute inflammation; 2, focal mild acute inflammation; 3, severe acute inflammation with multiple crypt abscesses and/or focal ulceration; and 4, severe acute inflammation with multiple crypt abscesses, epithelial loss, and extensive ulceration. *p < 0.05 (unpaired Student’s t-test). **(C)** Percentage of neutrophil granulocytes in peripheral blood following induction of colitis by IgG+/IgM+ bacteria in C56BL/6J Mice. Ns (P>0.05); * (P<0.05); ** (P<0.01).

The findings presented in **Figures 3A–C** demonstrate that IgG+/IgM+ bacteria associated with SCZ significantly exacerbated colon pathology in C56BL/6J mice compared with IgG-/IgM- bacteria or the NaCl control group. As shown in **Figure 3A**, the pronounced shortening of colon length in the IgG+/IgM+ group indicates severe inflammation and tissue damage, which are hallmarks of colitis. This observation is further supported by the histopathological scores and staining shown in **Figures 3B, C**, where mice gavaged with IgG+/IgM+ bacteria exhibited higher scores, extensive inflammation, crypt abscesses, epithelial loss, and ulceration, whereas the IgG-/IgM- group showed no detectable inflammatory changes. Additionally, **Figure 3D** shows a notable decrease in peripheral blood neutrophil granulocytes counts in the IgG+/IgM+ group, which may reflect their recruitment to the inflamed colon or an altered immune response. Together, these results highlight the critical role of IgG+/IgM+ bacteria in driving colitis-like pathology and suggest that these bacterial subsets may contribute to intestinal inflammation observed in scizophrenia. Further studies are warranted to elucidate the underlying mechanisms and identify potential therapeutic interventions targeting these pathogenic taxa.

## 4 Discussion

In this study, we investigated the composition and characteristic of IgG/IgM-coated intestinal microbiota to determine whether Ig isotypes coatings are associated with microbial taxa capable of eliciting inflammatory responses that may contribute to disease pathogenesis. Our findings revealed that the highly IgG/IgM-coated fraction of the intestinal microbiota represents a distinct sunset with the potential to mediate inflammation and drive disease progression.

To characterize the intestinal immune response towards gut microbiota, we employed bacterial cell sorting based on IgG/IgM coating, followed by 16S rRNA gene sequencing. This approach is consistent with recent studies that have utilized magnetic-activated cell sorting (MACS) and 16S rRNA sequencing to profile immunoreactive components of the gut microbiota (34) and to assess microbial shifts in response to treatments (35). Our results demonstrate that this strategy provides an effectively means of categorizing intestinal bacteria based on their interactions with and recognition by the host immune system.

### 4.1 IgM-coated bacteria: association with disease severity

IgM, the first antibody mobilized during humoral immunity (36), can translocate across the mucosal epithelium via the polymeric immunoglobulin receptor (pIgR) (16) and provide early mucosal protection before secretory IgA predominates. Interestingly, pathogens such as *Toxoplasma gondii*, *Trypanosomatidae, and Plasmodium falciparum* have evolved IgM receptors (37), potentially facilitating immune evasion.

In our study, we observed a progressive increase in the abundance of highly IgM-coated bacteria from healthy controls to patients with other psychiatric disorders (OPD) and finally to patients with SCZ, suggesting a correlation between IgM-coated bacteria and disease severity. Thus, the extent of IgM-coating may serve as a potential biomarker of psychiatric disease activity. Supporting this notion, gnotobiotic mice colonized with highly IgM-coated bacteria exhibited distinct pathological features, including reduced peripheral neutrophil depletion, mild colon shortening, and mucosal inflammation, compared with mice receiving low- or uncoated bacteria. These effects may result from specific bacterial surface antigens that are recognized by the host’s B1 cells, leading to the production of large amounts of specific IgM and the formation of a “high-IgM coating”. Such labeled bacteria can continuously activate B cells, driving clonal expansion of IgM-positive B cells (38). Activated B cells contribute a pro-inflammatory gut environment through antibody secretion, cytokine production and interactions with T cells (39). The ensuing inflammation leads to mucosal damage and colon shortening. Simutaneously, the inflammatory signals promote massive recruitment of neutrophils from the bloodstream into the intestinal tissue, resulting in a significant decrease in their counts in the peripheral blood (40). However, anxiety-like behaviors did not differ significantly across transplantation groups possibly because immune activation in this model did not affect the key brain regions involved mood and anxiety regulation (41), or because the specific neuroimmune pathways underlying behavioral modulation were not activated (42). Our future work will aim to identify specific neurobehavioral targets of inflammation through a multifaceted approach, including quantification of systemic inflammation, assessment of brain immune status, neurochemical profiling of key brain regions, and evaluation of multiple behavioral domains as Xin Pan.et al (43).

Taxonomic analysis revealed that highly IgM-coated bacteria were primarily composed of *Rhodococcus, Shigella, Clostridium*, and *Streptococcus* species. These microbes possess invasive structural features: *Clostridium* bears peritrichous flagella, whereas *Shigella* and *Streptococcus* express fimbriae, which facilitate mucus penetration and epithelial adhesion, thereby exacerbating mucosal damage. These findings are consistent with previous studies reporting *Clostridium* depletion in celiac disease (44), *Pseudomonas* and *Streptococcus* associations with cognitive impairment (45, 46), and *Escherichia-Shigella* abundance in generalized anxiety disorder (47). Collectively, these results underscore the importance of elucidating how highly IgM-reactive bacteria contribute to neuropsychiatric pathophsiology.

### 4.2 IgG-coated bacteria: a potential protective role?

Our study revealed a significant correlation between IgG-mediated immune responses and schizophrenia pathophysiology. We observed a progressive decline in the abundance of IgG-coated bacteria from healthy controls to patients with OPD and schizophrenia. Alpha -diversity analyses showed markedly reduced richness, phylogenetic diversity, and evenness of highly IgG-coated bacteria in patients with schizophrenia. While studies directly examining the relationship between IgG and the gut microbiota remain limited, research focusing on immune activation subtypes and gut microbiome function provides valuable insights. Emerging evidence indicates that a distinct subgroup of patients with schizophrenia (approximately 46.5%) exhibits a peripheral immune activation phenotype which characterized by a unique gut microbial signature, distinguishing them from both healthy controls and other patients without pronounced immune dysregulation (48).

Operational taxonomic unit (OTU) analysis revealed a significant depletion of highly IgG-coated bacteria in schizophrenia, particularly affecting *Rhodococcus, Acinetobacter*, and *Pseudomonas*. These taxa have concerning the pathogenic potential of these taxa lies in the fact that *Acinetobacter* is an opportunistic, multidrug-resistant pathogen, whereas *Pseudomonas* is notorious for causing nosocomial infections. Intriguingly, mice colonized with highly IgG-coated bacteria developed pathological changes resembling those induced by highly IgM-coated bacteria; however, the inverse association between IgG coating levels and disease severity suggests a potential protective role of IgG-coated bacteria in mental health contrasting with the pro-inflammatory role of IgG observed in chronic enteropathies (30, 34, 49). This discrepancy may be explained by variation in IgG glycoforms, rather than total IgG levels alone. This means that a reduction in protective IgG glycoforms (e.g., highly fucosylated forms) combined with a relative increase in pro-inflammatory types of IgG, such as afucosylated IgG (50). may influence on the development and severity of mental disorders. Future studies should further investigate the role of IgG subclass distribution and glycosylation characteristics in schizophrenia to clarify their roles in disease mechanisms.

### 4.3 Microbiota-mediated neutrophil modulation

In addition to inducing intestinal inflammation, transplantation of highly IgG/IgM-coated bacteria significantly reduced peripheral blood neutrophil percentages compared with transplantation of low-coated bacteria. The underlying mechanisms remain unclear but may involve microbial-derived neutrophil modulators (51). The gut microbiota produces both activators (e.g., peptidoglycan via NOD1 signaling (52) and LPS as a chemoattractant (53) and inhibitors (e.g., enterobactin suppressing ROS and NET generation (54), suggesting bidirectional neutrophil regulation. Thus, microbial rebalancing may represent a promising therapeutic strategy for correcting neutrophil dysregulation in inflammatory and neuroimmune diseases.

### 4.4 Study limitations

This study has several limitations. First,16S rRNA gene analysis provides limited taxonomic resolution, preventing species-level characterization. Second, the small sample size constrained our ability to perform stratified analyses by clinical variables. Third, we did not explore additional behavioral domains to clarify the negative results obtainein in the behavioral tests. Future studies with larger cohorts, metagenomic sequencing, and extension to other neurological domains are needed to validate these findings.

## 5 Conclusion

Our MACS-based profiling of immunoglobulin subtypes substantial population of IgG/IgM-coated bacterial capable of inducing intestinal in flammation in patients with schizophrenia. These findings not only highlight associations between specific bacteria and disease activity but also indicate that targeted microbial interventions may represent a promising therapeuticstrategy for schizophrenia. Further research should aim to clarify the mechanistic relationships among immunoglobulin-coated microbiota, gut inflammation, and neuropsychiatric symptoms to advance toward precision microbiome-based therapies.

## Data Availability

The original contributions presented in the study are publicly available. This data can be found here: China National GeneBank DataBase (CNGBdb), accession CNP0008779.
